# Prioritizing Wetlands for Waterbirds in a Boom and Bust System: Waterbird Refugia and Breeding in the Murray-Darling Basin

**DOI:** 10.1371/journal.pone.0132682

**Published:** 2015-07-10

**Authors:** Gilad Bino, Richard T. Kingsford, John Porter

**Affiliations:** 1 Centre for Ecosystem Science, School of Biological, Earth and Environmental Sciences, University of New South Wales, Sydney, NSW, Australia; 2 Ecosystem Management Science, NSW Office of Environment and Heritage, Sydney, NSW, Australia; University of Southern California, UNITED STATES

## Abstract

Dryland rivers have considerable flow variability, producing complex ecosystems, processes, and communities of organisms that vary over space and time. They are also among the more vulnerable of the world’s ecosystems. A key strategy for conservation of dryland rivers is identifying and maintaining key sites for biodiversity conservation, particularly protecting the quantity and quality of flow and flooding regimes. Extreme variability considerably challenges freshwater conservation planning. We systematically prioritised wetlands for waterbirds (simultaneously for 52 species), across about 13.5% of the Murray-Darling Basin (1,061,469 km2), using a 30-year record of systematic aerial surveys of waterbird populations. Nine key wetlands in this area, primarily lakes, floodplains, and swamps, consistently contributed to a representation target (80%) of total abundances of all 52 waterbird species. The long temporal span of our data included dramatic availability (i.e., booms) and scarcity (i.e., busts) of water, providing a unique opportunity to test prioritisation at extremes of variation. These extremes represented periods when waterbirds were breeding or concentrating on refugia, varying wetland prioritisation. In dry years, important wetlands for waterbirds were riverine and lacustrine (12 wetlands) but this changed in wet years to lacustrine and palustrine (8 wetlands). Such variation in ecosystem condition substantially changes the relative importance of individual wetlands for waterbirds during boom and bust phases. Incorporating this variability is necessary for effective conservation of Murray-Darling Basin waterbirds, with considerable generality for other similarly variable systems around the world.

## Introduction

Dryland rivers constitute more than a third of the total length and discharge of the global river network [1]. In Australia, about 70% of rivers are intermittent, predominantly dryland, systems [2, 3]. The Australian dryland rivers exhibit the highest flow variability in the world [4], driven primarily by the El Niño Southern Oscillation (ENSO) phenomenon [5, 6]. ENSO phases tend to last 1–2 years and recur every 3–8 years [7]. Flow variability is a key driver of the system, affecting water quality [2], biogeochemical processes [8] and community composition of organisms [9–11]. This flow variability drives ‘boom’ and ‘bust’ phases, affecting flooded habitats, processes and abundance, distributions, recruitment and habitat use of aquatic organisms [12–19].

Dryland rivers are also among the more vulnerable global ecosystems, given burgeoning human populations and increasing water scarcity [20, 21]. Altered landforms, geomorphological processes and natural flow regimes have driven degradation, affecting connectivity and diminishing the frequency, extent, and quality of available habitats [21–24]. Global freshwater biomes are possibly degrading faster than terrestrial or marine biomes [25]. To mitigate such threatening processes, biodiversity conservation strategies have strongly focused on protected areas [26], with assumed protection of habitats and critical ecosystem processes supporting biodiversity. Freshwater protected areas have often failed (e.g. [27]) because protection of critical water resources outside the area boundaries is often not possible. They also often fail to maintain connectivity for dependent aquatic species [28, 29]. Threats need to be mitigated at appropriate scales [30, 31]. Conservation efforts can try to maintain the quantity and quality of flow regimes [32–34] or rehabilitate systems by recovering water as environmental flows, altering dam operations, managing protected areas, and effective governance and adaptive management [35, 36]. For large river basins, prioritisation of wetlands for environmental water and protected areas remains a critical challenge.

Prioritising areas for conservation is underpinned by quantitative and systematic processes, attempting to capture biodiversity and landscape complexity [37, 38]. Schemes to prioritise conservation areas use fine-scale biological and environmental data for comprehensiveness, adequacy, representation, and efficiency [39], aiming to include the full range of species, processes and ecosystems. Systematic conservation planning approaches are increasingly applied to freshwater ecosystems [40–43], incorporating longitudinal connectivity [44] and condition [45]. Environmental flow management remains challenging to integrate [31]. The high spatiotemporal variability of boom and bust ecosystems, characterised by extreme variations in water availability [46–48], considerably challenges freshwater conservation planning. Capturing such variable biodiversity responses requires long-term ecological datasets at appropriate scales, implemented within a systematic conservation planning approach.

**Fig 1:**
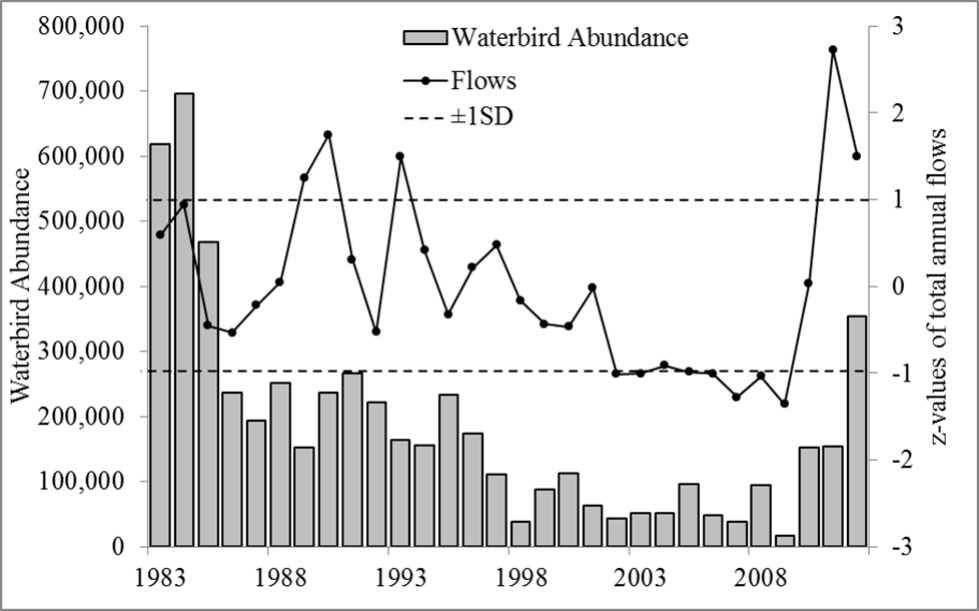
Total annual surveyed waterbird abundance, 1983–2012 (grey bars) and the corresponding z-values of total annual flows (dashed line indicated 1SD bounds) in the Murray-Darling Basin.

We aimed to prioritise wetlands for waterbirds using a systematic conservation planning approach, using a unique 30-year record of systematic aerial surveys of multispecies waterbird populations. The surveys systematically sampled 13.7% of the Murray-Darling Basin’s wetlands, with intervals of 200km between survey bands and placement based on random choice of an initial survey band (Kingsford and Porter 2009). Surveys intersected wetland types with similar proportions to those found across the basin. During this period, the Murray-Darling Basin experienced several climatic phases of boom and bust (Fig 1). Waterbird surveys [49, 50], tracked changes in populations and communities [27, 51–53], (Fig 1). Waterbirds are highly dependent on “boom” and “bust” phases of water availability [12, 17], using different freshwater ecosystems for feeding and breeding. They are a diverse vertebrate group, useful for monitoring and assessing freshwater ecosystems [17, 50, 54]. Prioritising wetlands that adequately represent the full breath of waterbird diversity may significantly improve conservation outcomes, over simple assessment of total numbers or diversity. The large temporal span of our data provided a unique opportunity to test how prioritisation of wetlands varied across years and how prioritisation of wetlands changed with water availability.

## Methods

### Aerial Surveys

**Fig 2:**
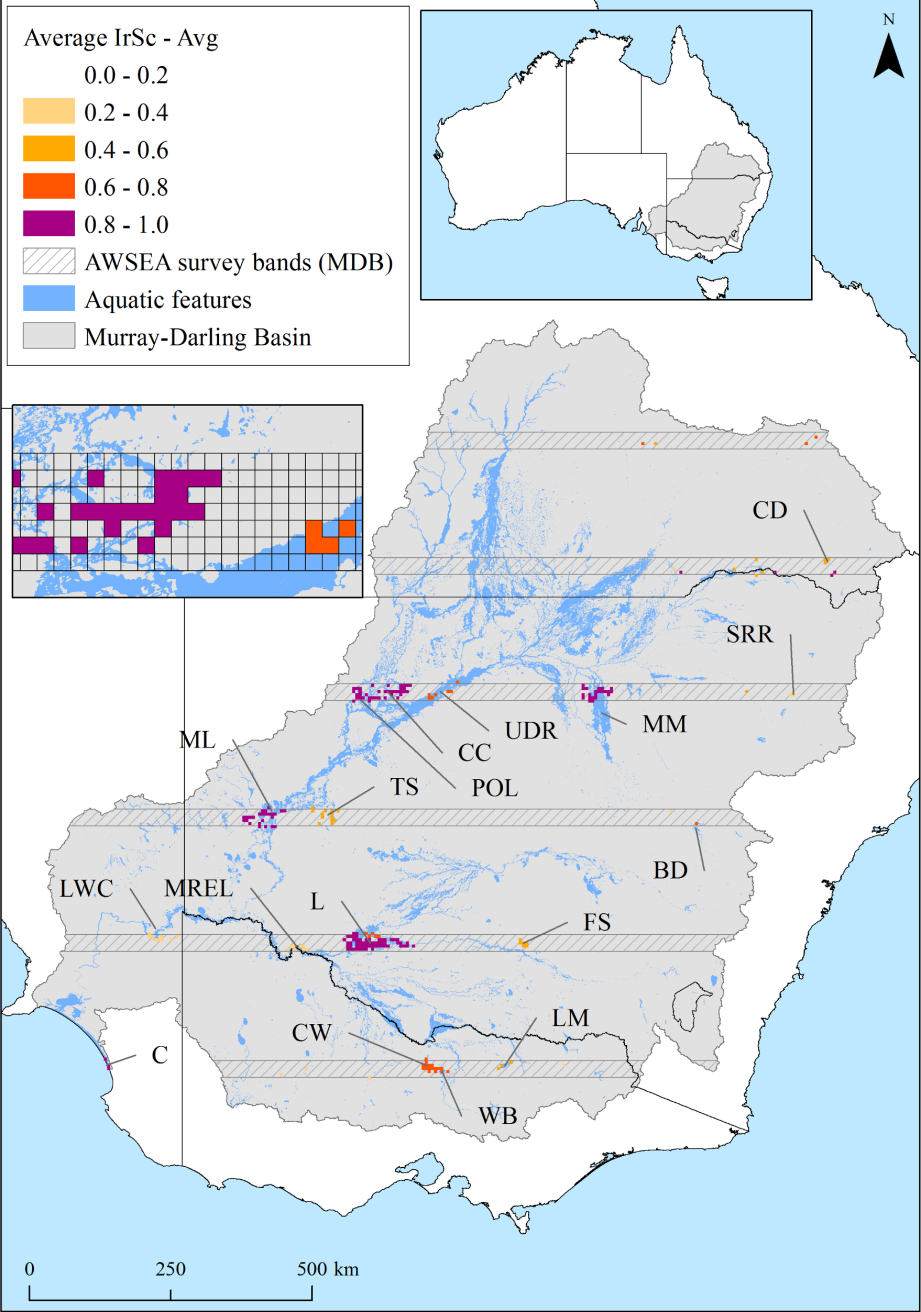
Average irreplaceability scores (IrSc) of planning units (PUs) (small inset) across increasing prioritisation targets (10–100%) of total waterbird abundance (1983–2012), for the 52 waterbird species (see Table 2 for wetland acronyms). The six (30km wide) aerial survey bands sampled 13.7% of the wetland surface area of the Murray-Darling Basin (inset, grey shade). Wetlands were mapped from satellite imagery (Kingsford et al. 2004).

We used multispecies waterbird count data from aerial surveys across eastern Australia, a large and long running wildlife survey in Australia (1983–2012) [50, 52]. This aerial survey has been shown to provide a cost-effective method for surveying large number of waterbirds across large areas [52]. An area of 2,697,000 km^2^ was systematically sampled across 10 survey bands, 30 km in width, spaced every 2° of latitude (~200 km) from 38°30’S to 20°30’S [55, 56]. Seven of these survey bands covered the Murray-Darling Basin, about 13.5% (143,365 km^2^) of its land surface area (1,061,469 km^2^, Fig 2). The seven survey bands intersected with 13.7% (8,984 km^2^) of the total wetland area (65,585 km^2^), comprising dams and natural wetlands (i.e., estuarine (8.5%), lacustrine (10.9%), palustrine (14.2%), and riverine (12.5%)) [57]. The survey was considered an unbiased sample as proportions of the five natural wetland types were similar within the survey bands, compared to across all wetlands in the Murray-Darling Basin: estuarine (survey 0.45% cf. total 0.72%), lacustrine (9.6% cf. 12.0%), palustrine (87.9% cf. 85.0%), and riverine (2.1% cf. 2.3%). Total area of mapped reservoirs in the Murray-Darling Basin was 1,154 km^2^ (1.76%) of which 238 km^2^ were surveyed, representing 2.73% of surveyed wetlands.

Fifty two waterbird species or grouped taxa were identified and counted on all wetlands >1ha and on small wetlands on an ad hoc basis, within survey bands (Fig 2 and S1 Table). On average, 351±157.7 (range 116–710) wetlands were surveyed each year.

### Prioritisation

For prioritising wetlands for waterbird representation, we used the Marxan software [58]. It uses a simulated annealing algorithm across a set of planning units, capturing defined targets for a minimum total cost [59]. We solved a minimum-set problem by setting an objective function that minimised costs for these defined targets. Our costs were number of wetlands while representations targets were a proportion of total abundance for each of the 52 waterbird species over all wetlands. One challenge was the different boundaries of wetlands and floodplains. We divided the land surface of the Murray-Darling Basin into 0.05x0.05 degree planning units (PUs) (~5x5km), selecting the 7,202 PU that intersected aerial survey bands (Fig 2). Our decision on using relatively small and equally-sized PUs was made due to the large temporal variation in size of wetlands and floodplain. We then assigned each PU to its proximate wetland, identified using the national 1:250,000 waterbody layer [57] and records made during the aerial survey. Small wetlands, particularly farm dams do not appear on this mapping, although they were surveyed. These were assigned to the intersecting planning unit, even though they did not intersect with a mapped wetland. Only a small proportion of waterbirds are found on these small wetlands (Kingsford and Porter 2009). Generally the natural wetlands are large and distinctive. Where several PUs intersected a large wetland\floodplain, we aggregated those PUs into a single wetland entity (hence a single PU). For each of the final 1,316 PUs, we summarised the total abundance for each waterbird species stratified according to survey year (1983–2012, n = 30). We set a constant cost for each PU, to identify minimal sets of PUs that achieved our conservation targets. We examined wetland prioritisation scenarios with an incremental (10%) increase in representation targets (10–100%) for total abundance of each waterbird species, simultaneously across all species (52 species, 1983–2012). We calculated the rate of increase in number of planning units, with increasing representation targets to identify the most cost-effective representation target. Based on the identified representation target, we then prioritised all PUs for each of the 30 years separately, based on annual waterbird counts. For each scenario (i.e., varying representation targets and 30 years), we ran 1000 solutions identifying PUs frequently selected across all solutions, a measure of irreplaceability for each year [60]. We then estimated the irreplaceability scores (IrSc) for each PU (range 0–1), where scores close to one represented PUs critical for achieving representation target while scores near zero represented PUs that did not contribute to required target. For ease of reporting, we categorised IrSc into five classes: very low (IrSc<0.2), low (0.2≤IrSc≥0.4), moderate (0.4≤IrSc≥0.6), high (0.6≤IrSc≥0.8), and very high (0.8≤IrSc≥0.99) and completely irreplaceable (IrSc = 1) [61, 62].

We then examined two aspects of annual variation across the basin: (1) total number of PUs, required to achieve representation targets, and (2) IrSc of each PU, across the entire basin over the 30 years. We modelled these variations, using a generalized linear model, against annual estimates of water availability in the Murray-Darling Basin and in other parts of the Australian continent. We incorporated estimated flow, rainfall and Southern Oscillation Index (SOI) which can be directly or indirectly related to wetland and resource availability of waterbirds occurring across the continent [12, 63, 64]. For rainfall, we compiled total annual rainfall for the Murray-Darling Basin, Lake Eyre Basin, South-Eastern region (Melbourne); Eastern region (Brisbane); Northern region (Darwin); and Southern region (Adelaide) [65]. We obtained monthly Southern Oscillation Index [65] and calculated an annual estimate, as phases are tied to El Nino and La Nina cycles associated with major periods of flooding and drying in Australia [19, 66]. We used total annual flows in the Murray-Darling Basin (logged transformed) [67].

We scaled all predictor variables to easy comparison. We tested for collinearity among predictor variables using the variance inflation factor (VIF) of each predictor[68]. We used a conservative measure, with a collinearity threshold of VIF≥5. Subsequently, we removed total rainfall from the Southern and Eastern regions from analyses. To deal with the uncertainty around the direct contribution of water availability in the Murray-Darling Basin, we used a Bayesian Model Averaging (BMA) approach for generalised linear models, assuming Gaussian errors for total number of annual PUs and Poisson errors for IrSc of each PU. We used the ‘bic.glm’ in the ‘BMA’ package [69], within the R statistical environment [70]. The BMA for generalised linear models uses the ‘leaps and bounds’ algorithm (Furnival and Wilson 1974) and a weighted averaging algorithm, based on Bayes’ theorem, with weights proportional to the approximate posterior model probability to represent the relative strength of evidence in favour of each model [71, 72]. As a measure of goodness-of-fit, we calculated Efron’s pseudo R^2^ (squared correlation between the predicted values and actual values) [73]. The BMA process enabled us to estimate the posterior probability of inclusion of water availability as a driver of annual variation in: 1) total number of PUs and 2) the IrSc in each PU. For the latter, we limited analysis and interpretation to PUs with IrSc in at least five years and quantified the relationship (i.e., negative or positive) by estimating the posterior mean coefficient of annual flows.

We used estimates of total annual flows across the Murray-Darling Basin to identify years experiencing dry and wet conditions respectively: the bottom 25 percentile (2003, 2005–2009) and the top 75% (1984, 1988–1990, 2010–2012). We then compiled IrSc of PUs, under these extreme dry or wet years and examined how PU targets varied. Within the space of IrSc under dry and wet years, we defined wetlands according to their potential ecological function: refugia (PUs with high IrSc (≥0.6) in only dry years), breeding (PUs with high IrSc (≥0.6) in only wet years), and both function (PUs with high IrSc (≥0.6) in both dry and wet years). This was rationalized since waterbird breeding is positively related to flow and rainfall while during times of low water availability waterbird seek refuge in semi-permanent and permanent water bodies [74–76]. Cutoffs provided summaries, rather than representing hard boundaries of ecological function.

We also tested for variation in waterbird occupancy of wetland types (measured as IrSc), between dry and wet years. To do this, we used established classifications of estuarine, lacustrine, palustrine, and riverine wetlands [57]. Wetland types represented distinct habitats, driven by unique hydrological regimes, functionally different in the ecology of waterbirds, providing opportunities for feeding and breeding [17]. We then calculated the summed IrSc of each wetland type, corrected by total wetland area, and computed the proportion of irreplaceability under dry and wet years. To support any sign for significant differences, we performed a Pearson's Chi-squared Test using the built-in “chisq.test” function within the R statistical software [70].

## Results

### Prioritising for waterbird communities

The number of wetlands required for different representation targets (10–100%) of total abundance, 1983–2012, increased at varying rates. Representation of 10% of abundance for each species was achieved with only 4 wetlands (IrSc≥0.6): Lowbidgee, Cuttaburra Channels, Lower Coorong, and the Thallon Waterholes, which also encompassed 54% of Banded lapwing (*Vanellus tricolor*) and 13% of Wandering whistling-duck (*Dendrocygna arcuata*) abundance (Fig 2 and S2 Table). Respective representation increments of 10% increased the number of identified wetlands to: 6, 8, 11, 16, 20, 27, 34, 56, and 225. Additional priority wetlands were consistently identified (average IrSc≥0.6) with increasing representation targets: Paroo Overflow Lakes, Menindee Lakes, Macquarie Marshes, Waranga Basin (51% of total Silver gull (*Larus novaehollandiae*) abundance), and Bracker Creek (71% of total Magpie goose (*Anseranas semipalmata*) and 19% of total Plumed whistling-duck (*Dendrocygna eytoni*) abundance), (Fig 2). Incremental increase in representation targets highlighted two cost-effective targets: from 80% to 90% and 90% to 100%, requiring respective increases of 65% and 302% in the number of wetlands.

### Temporal variation

**Fig 3:**
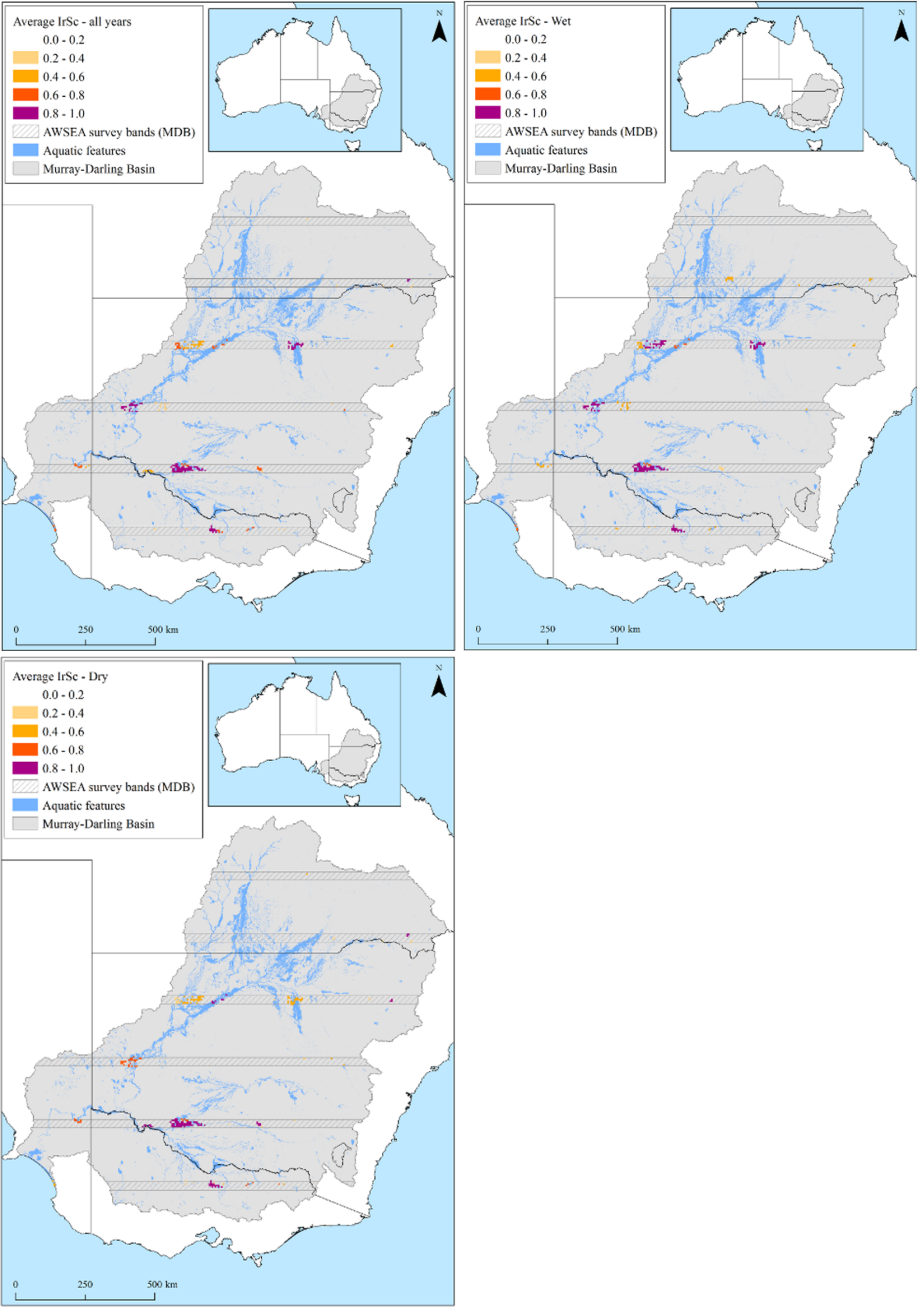
Average irreplaceability scores, when prioritising for a representation target of 80% of waterbird abundance for each of the 52 waterbird species, simultaneously across all species and (A) across all years 1983–2012; (B) during wet years; and (C) during dry years.

Annual estimates of waterbirds across the Murray-Darling Basin (1983–2012) varied considerably from 17,171 to 695,781 waterbirds in the surveyed area (mean = 186,050, 95%CI: 127,398–244,701) (Fig 1). Over the 30-year period, three PUs averaged 0.8≤IrSc≤1 (Lowbidgee, Corop Wetlands, Menindee Lakes) and eight PUs averaged 0.6≤IrSc≤0.8 (Macquarie Marshes, Coolmunda Dam, Fivebough Swamp, Paroo Overflow Lakes, Lower Coorong, Upper Darling River, Cuttaburra Channels, and Waranga Basin) (Fig 3A). The number of PUs required over this 30-year period to achieve a representation target of 80% for all 52 waterbird species, ranged from 13 PUs (IrSc≥0.6) (1986) to 47 PUs (1999), (average PUs 26.3, 95%CI: 23.34–29.25). Total flows in the Murray-Darling basin had largest negative effect the (β = -1.57, posterior probability of inclusion *pp = 0*.*53*) on the number of PUs required to achieve a representation target of 80%, meaning the number of PUs decreased with increasing flows (Table 1). There was also some indication of increasing number of PUs with years (β = 0.66, *pp* = 0.29).

**Table 1:** Results of generalized linear model analysis, using Bayesian model averaging, showing the posterior probability (pp) and posterior mean coefficient estimates (β±sd) for explanatory variables (scaled), (Pseudo R2 = 0.23). Analyses focused on numbers of planning units (PUs) required, each year (1983–2012), to achieve an 80% representation target of abundance for each of the 52 waterbird species, simultaneously across all species, across the surveyed Murray-Darling Basin.

Explanatory Variable	pp	β±sd
Intercept	1.000	26.33±1.33
Flow–Murray-Darling Basin	0.532	-1.574±1.864
Year	0.294	0.655±1.382
Rainfall–South-eastern Australia	0.277	-0.585±1.286
Rainfall–Northern Australia	0.246	0.549±1.434
Rainfall—Lake Eyre Basin	0.218	-0.453±1.404
Southern Oscillation Index	0.111	0.068±0.566

**Fig 4:**
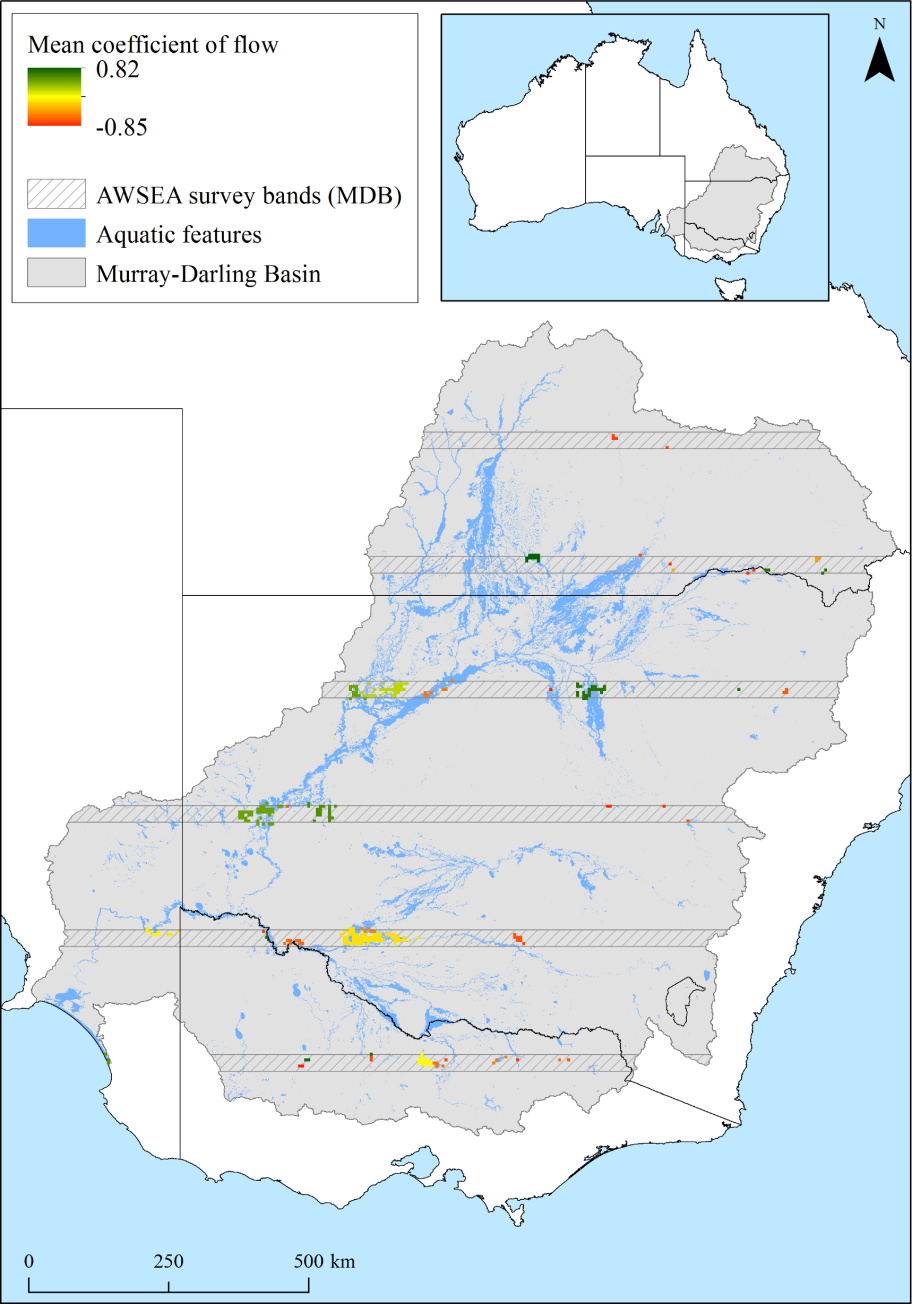
Posterior mean coefficient (estimated using Bayesian Model Averaging) of total annual flows in the Murray-Darling Basin, against irreplaceability scores (IrSc) of each planning unit (PU), 1983–2012.

There was also spatial variation in which PUs were included each year, reflecting the size of annual river flows across the basin, 1983–2012. Forty PUs had annual flows in the Murray Darling Basin with a posterior probability (pp) ≥0.6 (Pseudo R^2^ = 0.25±0.12sd). Of these, 17 had a positive relationship with annual flows while 32 were negatively related to annual flows (Fig 4). Noteworthy wetlands with strong positive relationship included: Macquarie Marshes (mean posterior coefficient = 0.28), Tallywalka system (0.23), Lower Coorong (0.23), Paroo overflow lakes (0.15), Menindee Lakes (0.15), Cuttaburra Channels (0.14), and Corop Wetlands (0.10) (Fig 4). Noteworthy wetlands with negative relationship included: Fivebough Swamp (-0.27), Split Rock Reservoir (-0.24), Burrendong Dam (-0.19), Murray River and Euston Lakes (-0.15), Waranga Basin (-0.11), and Upper Darling River (-0.10) (Fig 4).

**Fig 5:**
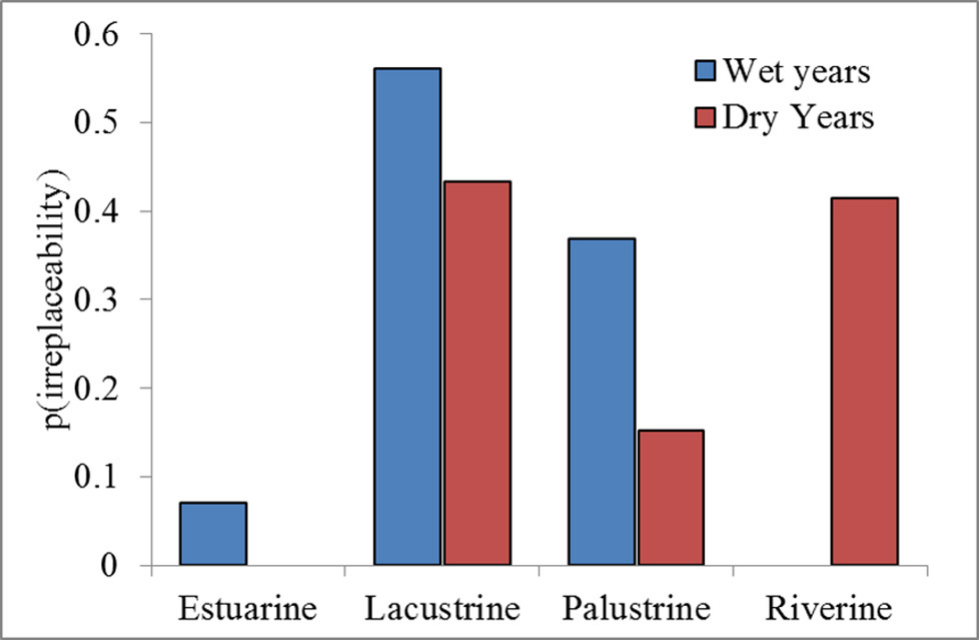
Proportion of irreplaceability scores by system type (estuarine, lacustrine, palustrine, riverine), during wet and dry years.

**Fig 6:**
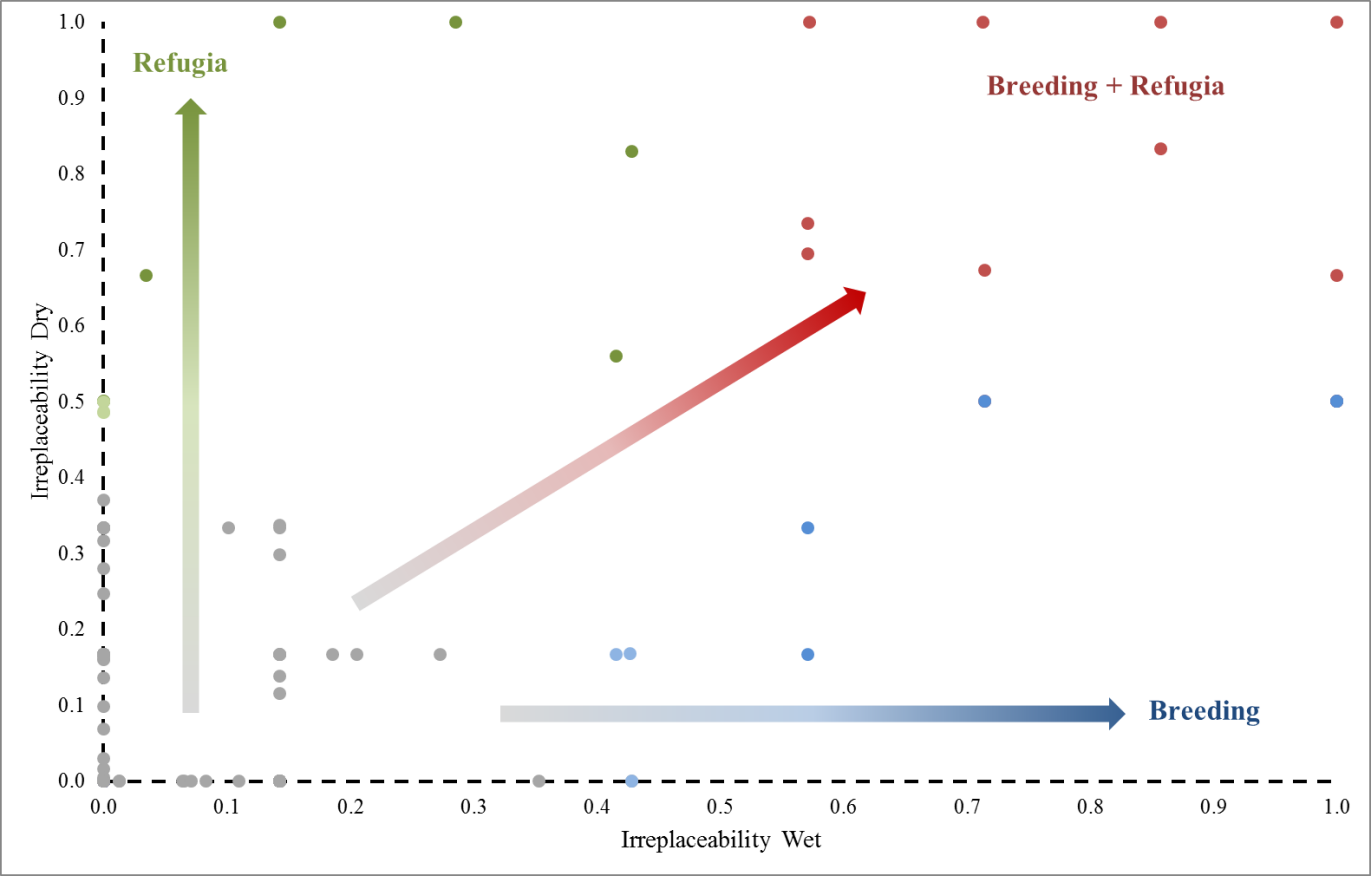
Irreplaceability scores of PUs under wet and dry phases, showing arrows that indicate functional role of wetlands as solely as refugia or, breeding sites and wetlands supporting both functions.

Significant differences were apparent between wetland types (estuarine, lacustrine, palustrine, and riverine) and their prioritisation (IrSc), varying across wet and dry years (χ^2^ = 201.3, df = 3, p<0.001), (Fig 5). In dry years, PUs in riverine and lacustrine systems had high IrSc, while in wet years, lacustrine and palustrine were important. In wet years (i.e., ≥75% percentile Murray Darling Basin annual flows), number of PUs for species-level representation ranged between 14 (2012) to 21 (1989), (average 17.57, 95%CI: 15.71–19.43), (Fig 3B). Two PUs had an average IrSc = 1 (Lowbidgee, Cuttaburra Channels); three PUs averaged 0.8≤IrSc≤1 (Macquarie Marshes, Corop Wetlands, Menindee Lakes) and; three PUs averaged 0.6≤IrSc≤0.8 (Lower Coorong, Paroo Overflow Lakes, Waranga Basin (62% of Silver Gull and 9% Chestnut Teal (*Anas castanea*) abundances). In dry years (i.e., <25% percentile Murray-Darling Basin annual flows), the number of PUs ranged between 16 (2008) and 42 (2007) for 80% representation (average 27.33, 95%CI: 19.97–34.70), (Fig 3C). Two PUs had an average IrSc = 1 (Coolmunda Dam, Fivebough Swamp), four PUs with average 0.8≤IrSc≤0.99 (Lowbidgee, Upper Darling River, Corop Wetlands, Waranga Basin (16% of Chestnut Teal and 12% Silver Gull abundances)), and six PUs averaged 0.6≤IrSc≤0.8 (Murray River and Euston Lakes, Split Rock Reservoir, Lake Mokoan, Lindsay-Walpolla-Chowilla Wetland Complex, Burrendong Dam, Great Cumbung Swamp). Wetlands that were important only in wet years and not dry years included the Cuttaburra Channels (1 wet/0.47 dry), Menindee Lakes (0.96 wet/0.44 dry), Macquarie Marshes (0.95 wet/0.42 dry), Lower Coorong (0.71 wet/0.5 dry), and Paroo Overflow Lakes (0.6 wet/0.22 dry). Contrastingly, refugia were represented by wetlands important in dry and not wet years (Fig 6). These included Coolmunda Dam (0.47 wet/1 dry), Fivebough Swamp (0.27 wet/1 dry), Upper Darling River (0.58 wet/0.89 dry), Murray River and Euston Lakes (0.14 wet/0.79 dry), Split Rock Reservoir (0.37 wet/0.72 dry), Lake Mokoan (0.47 wet/0.71 dry), Lindsay-Walpolla-Chowilla Wetland Complex (0.40 wet/0.70 dry), Burrendong Dam (0.24 wet/0.62 dry). Three wetlands were important in both wet and dry conditions (Fig 6): the Lowbidgee (1 wet/0.94 dry), Corop Wetlands (0.86 wet/0.85 dry), and Waranga Basin (0.76 wet/0.81 dry).

## Discussion

Clear differences emerged in the analysis of wetlands identified for waterbird conservation across the Murray-Darling Basin, based on 30-years of aerial surveys. Several key wetlands consistently represented the full breadth of waterbird diversity (S2 Table). These were generally large natural wetlands, usually lakes, floodplains and swamps (Table 2). Importance of wetlands varied between years and in response to water availability (Figs 1 and 3–5). Such spatiotemporal variation in wetland importance exemplifies the considerable challenges in developing a conservation planning framework for the protection of wetland ecosystems, across large scales, when there are few data. This problem is particularly acute for dryland river systems which experience the highest levels of temporal flow variation in the world [77]. Capturing the spatiotemporal dimensions in a boom and the bust system is essential for effective conservation of biodiversity dependent on such rivers and wetlands, because long-term viability of the many organisms are highly reliant on such dynamics (Figs 3 and 6). Important habitats need to be protected for boom and bust phases. Despite this challenge, there were still relatively few key wetlands of importance during boom and bust phases.

**Table 2:** Wetland complexes in the Murray-Darling Basin identified as priorities when setting an 80% representation targets for each of the 52 waterbird species, simultaneously across all species, across all years (overall), during dry and wet years, along with total and average (±95% CI) waterbird abundances estimated during the aerial surveys of waterbirds in eastern Australia.

Wetland complex	Dry	Wet	Overall	Average, 95%CI
Lowbidgee	X	X	X	52728, 32628–72828
Cuttaburra Channels		X	X	20399, 6439–34359
Menindee Lakes		X	X	15551, 4735–26367
Macquarie Marshes		X	X	11426, 3626–19225
Paroo Overflow Lakes		X	X	10425, 4691–16159
Darling River	X		X	6836, -2402-16073
Corop Wetlands	X	X	X	5932, 3619–8246
Coorong, Lower Lakes and Murray Mouth		X	X	4011, 1109–6913
Fivebough Swamp	X		X	3940, 2512–5368
Coolmunda Dam	X		X	3814, 2498–5130
Great Cumbung Swamp	X			3730, 1524–5936
Burrendong Dam	X			2222, 50–4393
Murray River and Euston Lakes	X			2117, -175-4409
Waranga Basin^†^	X	X	X	1807, 424–3190
Lindsay-Walpolla-Chowilla Wetland Complex	X			1544, 639–2449
Lake Mokoan	X			1294, 808–1780
Split Rock Reservoir	X			970, 551–1389

† Likely driven by high occurrence of Silver gull

### Boom phases—breeding

Breeding habitats are important to identify because successful recruitment underpins long-term maintenance of populations (Fig 6). Breeding of waterbirds is triggered by large flooding events [36, 50, 75], potentially phased for different species [17]. These events substantially increase the viability of colonially breeding waterbirds and may catalyse successive breeding attempts [56, 76, 78, 79]. These floods, triggered by high flows produced high widespread aquatic productivity and considerable reproduction and recruitment of aquatic organisms [80–83]. Many of these provide resources for waterbirds. These large flows also maintain longitudinal (in-stream) and lateral (floodplain) connectivity [48, 84, 85], allowing for exchange of abiotic and biotic components between channel and floodplain environments [86].

Lacustrine and palustrine habitats were consistently important for waterbirds during boom years of high flows (Fig 5). These included eight wetland complexes, critical for supporting waterbirds during wet years (Table 2 and Fig 6). Of these, five were important during wet but not during dry years. These were: Cuttaburra Channels, Menindee Lakes, the Macquarie Marshes, Paroo Overflow Lakes, and Lower Coorong. The Macquarie Marshes are a well-known and critically important site for breeding waterbirds of high conservation importance [36, 87] and similarly, the Paroo wetlands regularly support large numbers of waterbirds [50], often in large breeding concentrations [51, 88, 89]. Combined, these wetlands provided habitat for almost 22,000 per year (Table 2). They are clearly wetlands of considerable importance, reflected in their gazettal as protected areas and Ramsar sites. However, only about 10% of the Macquarie Marshes is included in the protected areas system while only some of the Paroo overflow lakes are part of Paroo-Darling National Park. Further accentuating the importance of the Macquarie Marshes, our surveys only cover about one third of the Macquarie Marshes in the north (30km survey band), meaning their value for waterbirds is significantly higher than we measured during our surveys, possibly three times higher [90]. The maintenance of flow and flooding regimes to these wetlands remains of paramount importance for their long-term viability. For the Macquarie Marshes, given its considerable reduction in size and area as a result of diversions upstream [90], increasing environmental flows and their effective management remains critically important for waterbird populations [36].

### Bust phases—refugia

There are potential bottlenecks of habitat during bust phases, defined by the magnitude, frequency, and duration of drying [14, 91], when there are relatively few large wetland habitats with water available, at large spatial scales [18]. During these bust phases, there is large-scale desiccation and fragmentation of freshwater habitats, reducing freshwater habitat availability [92, 93]. Dry phases can also drastically reduce populations, affecting community structure, and driving extinctions in local populations [94, 95]. They represent periods of considerable concern for organisms such as waterbirds which are highly dependent on availability of wetland habitat [75]. Drying is an important process for wetland ecosystems, maintaining habitat heterogeneity and controlling complementary biochemical processes [96] but also ultimately contributing to wetland productivity for waterbirds [97]. Waterbirds can access these refuges given their capabilities for long distance dispersal [17, 98, 99]; such refugia (i.e., waterholes, lakes, rivers) can provide resources to survive during dry years [18, 100]. The spatial distribution and size of refuge sites is vital for waterbirds to survive over such bust phases when mortality is high [12, 17].

Refugia sustain the capacity of organisms to increase population sizes when inevitable boom phases follow [93]. We identified a relatively small number of refugia (Fig 6), identified as lacustrine and riverine habitats with high irreplaceability scores (Fig 5). During dry times and after the boom phases, waterbirds often concentrate in large numbers on such available wetlands [56]. Most prominently, these included the Upper Darling River, Fivebough Swamp, Coolmunda Dam (Table 2). Identifying and ensuring such refugia habitats are maintained during dry phases is critical for conservation. Similarly, there is opportunity to provide environmental water to such sites to ensure longevity of wetland habitat for waterbirds in an otherwise dry matrix of wetlands. Large dams (e.g., Coolmunda, Burrendong, and Waranga Basin) provide some opportunities for waterbirds to survive although it is not clear whether such habitats provide sufficient food for long periods. Coolmunda Dam is a large shallow during dry periods, providing considerable edge habitats for waterbirds. These dams may become increasingly important as some large lakes and swamps on regulated rivers likely retain water less frequently than before river regulation, as drying periods have increased [101, 102]. Also as the dams become shallower, they may also become functionally more like natural wetlands.

### Management implications

Increased drying phases may push organisms, including waterbirds, beyond their limits of natural resilience, given compounding impact of river regulation and water abstractions [103, 104]. In the heavily regulated Murray-Darling Basin, identifying a subset of wetlands that can be managed with environmental water during dry periods may also be particularly important. This is particularly challenging for the basin which supports over 30,000 wetlands, including 16 of international importance [105]. Many of these wetlands have undergone significant degradation due to river regulation and modified hydrological regimes [101]. Recent acquisition of environmental flow allocations provides opportunities for recovery of some functions and increasing their functions as refugia [106, 107]. Watering actions commonly target specific wetlands, using available environmental water for key objectives (e.g. waterbird breeding) in a particular year. Developing a prioritisation framework, at large spatial and temporal scales, for wetlands could significantly improve allocation of environmental water at large spatial scale where particular refugia might be critical for waterbirds. For highly mobile waterbirds, this needs to be coordinated across wetlands, across river catchments, managed through boom and bust phases. This is also particularly important for waterbird breeding which may be critically important [76, 108]. Our analyses were limited by the coverage of only 13.7% of all wetlands in the Murray-Darling Basin [56], given long-term data availability. There is future opportunity to extend such analyses to all wetlands across this basin, identifying all key wetlands for waterbirds [109], with appropriate long-term data. We considered our sample of wetlands in the survey bands reasonably unbiased as survey bands were randomised and systematically placed across the Murray-Darling Basin (Fig 1). These survey bands covered the major rivers and different climatic zones. Further, there was similarity in the proportions of each wetland types surveyed, compared all wetlands across the Murray-Darling Basin. This provides some confidence that our wetland prioritisation can represent the full breath of waterbird diversity as well as the functions of different types of wetland in the Murray-Darling Basin, albeit requiring the data for other wetlands to show this.

### Challenges

Considerable challenges remain for developing a conservation approach that prioritises not only wetlands but also management strategies for waterbirds at the scale of an entire basin. Shortlisting which waterbird species are of conservation concern will narrow the number of wetlands. For example, Waranga Basin was identified as important because it held large concentrations of Silver gull, an acknowledged pest species [110], but would clearly be omitted. This could focus management on species at risk of extinction (e.g., Australasian bittern (threatened), painted snipe (threatened), Cape Barren goose (vulnerable)). Also, there is a need to incorporate understanding of population dynamics to assess the long-term viability of individual species [111]. This could identify vital requirements and support targeted environmental flow allocations. These would require compiling waterbird life histories, along with more fine-scale studies of behaviour and habitat requirements. Lower trophic levels would also require attention, such identifying the importance of food resources to different species (e.g. fish for piscivorous waterbirds [29, 31]. Dealing with migratory birds will also require protecting habitats and resources beyond the Murray-Darling Basin during the non-breeding season [112]. Large scale climatic events can also confound observed variation in waterbird abundance and composition, although recent work indicates water availability within the basin is the largest driver of waterbird abundances [113].

### Conclusions

Prioritising areas using long-term waterbird aerial surveys provided us with unique insights into the spatial and temporal variability of wetlands of importance for waterbirds during wet and dry extremes. Only a relatively small subset of wetlands provided habitat for a large proportion of recorded waterbirds (1983–2012), (Fig 6, Table 2). The long-term viability of waterbird species in highly regulated systems will ultimately depend on strategies that target key wetlands for conservation protection during natural boom and bust phases and reinstating these phases with environmental flows. In particular, it is important to ensure that sufficient refugia exist during dry periods to sustain waterbird populations and key breeding sites are a focus for environmental flow management.

## Supporting Information

S1 File Datasets used for the Marxan prioritisation process. (RAR)

S1 Table Fifty-two waterbird species identified during aerial surveys of waterbirds across the Murray-Darling Basin. (DOCX)

S2 Table Proportions of waterbird species found in identified key wetlands (Table 2) in all years (1983–2012), wet years (1984, 1988–1990, 2010–2012), and dry years (2003, 2005–2009). (XLSX)
