# Supplementary material for: Prioritizing Wetlands for Waterbirds in a Boom and Bust System: Waterbird Refugia and Breeding in the Murray-Darling Basin
Source: PLoS One. 2015 Jul 10;10(7):e0132682. doi: 10.1371/journal.pone.0132682 (PMC4498595; doi:10.1371/journal.pone.0132682)
Supplement: S1 Table — (DOCX) [file pone.0132682.s002.docx]

## Appendix 1. Fifty-two waterbird species identified during aerial surveys of waterbirds across the Murray-Darling Basin

| **Common name** | **Species name** |
| --- | --- |
| Australasian grebe | *Tachybaptus novaehollandiae* |
| Red-necked avocet | *Recurvirostris novaehollandiae* |
| Pacific black duck | *Anas superciliosa* |
| Banded lapwing | *Vanellus tricolor* |
| Pacific black duck | *Anas superciliosa* |
| Brolga | *Grus rubicundus* |
| Banded stilt | *Cladorhynchus leucocephalus* |
| Black swan | *Cygnus atratus* |
| Black-tailed native-hen | *Gallinula ventralis* |
| Australasian shoveler | *Anas rhynchotis* |
| Eurasian coot | *Fulica atra* |
| Caspian tern | *Hydroprogne caspia* |
| Chestnut teal | *Anas castanea* |
| Darter | *Anhinga melanogaster* |
| Small egrets^a^ |  |
| Intermediate egret | *Ardea intermedia* |
| Little egret | *Ardea garzetta* |
| Cattle egret | *Ardea ibis* |
| Freckled duck | *Stictonetta naevosa* |
| Gull-billed tern | *Sterna nilotica* |
| Great crested grebe | *Podiceps cristatus* |
| Glossy ibis | *Plegadis falcinellus* |
| Great cormorant | *Phalacrocorax carbo* |
| Small grebes^a^ |  |
| Hoary-headed grebe | *Poliocephalus poliocephalus* |
| Australasian grebe | *Tachybaptus novaehollandiae* |
| Grey teal | *Anas gracilis* |
| Plumed whistling-duck | *Dendrocygna eytoni* |
| Hardhead | *Aythya australis* |
| Black-necked stork | *Xenorhynchus asiaticus* |
| Little black cormorant | *Phalacrocorax sulcirostris* |
| Great egret | *Ardea alba* |
| Large wading birds^a^ |  |
| Eastern curlew | *Numenius madagascariensis* |
| Whimbrel | *Numenius phaeopus* |
| Little curlew | *Numenius minutus* |
| Bar-tailed godwit | *Limosa lapponica* |
| Black-tailed godwit | *Limosa nebularia* |
| Little pied cormorant | *Phalacrocorax melanoleucos* |
| Musk duck | *Biziura lobata* |
| Dusky moorhen | *Gallinula tenebrosa* |
| Masked lapwing | *Vanellus miles* |
| Australian shelduck | *Tadorna tadornoides* |
| Magpie goose | *Anseranas semipalmata* |
| Whiskered tern | *Sterna hybrida* |
| Nankeen night heron | *Nycticorax caledonicus* |
| Pied cormorant | *Phalacrocorax varius* |
| Pink-eared duck | *Malacorhynchus membranaceus* |
| Australian pelican | *Pelecanus conspicillatus* |
| Royal spoonbill | *Platalea regia* |
| Silver gull | *Larus novaehollandiae* |
| Purple swamphen | *Porphyrio porphyrio* |
| Small waders^a^ |  |
| Grey plover | *Pluvialis squatorola* |
| Lesser golden plover | *Pluvialis dominica* |
| Mongolian plover | *Charadrius mongolus* |
| Double-banded plover | *Charadrius bicinctus* |
| Black-fronted plover | *Charadrius melanops* |
| Red-capped plover | *Charadrius ruficapillus* |
| Ruddy turnstone | *Arenaria interpres* |
| Grey-tailed tattler | *Tringa brevipes* |
| Common sandpiper | *Tringa hypoleucos* |
| Marsh sandpiper | *Tringa stagnatilis* |
| Terek sandpiper | *Tringa terek* |
| Greenshank | *Tringa nebularia* |
| Red knot | *Calidris canutis* |
| Great knot | *Calidris tenuirostris* |
| Sharp-tailed sandpiper | *Calidris acuminata* |
| Red-necked stint | *Calidris ruficollis* |
| Curlew sandpiper | *Calidris ferruginea* |
| Broad-billed sandpiper | *Limicola falcinellus* |
| Red-kneed dotterel | *Erthrogonys cintus* |
| Latham's snipe | *Gallinago hardwickii* |
| Straw-necked ibis | *Threskiornis spinicollis* |
| Terns^a^ |  |
| Crested tern | *Sterna bergii* |
| Lesser crested tern | *Sterna bengalensis* |
| White-winged black tern | *Chlidonias leucopterus* |
| Australian wood duck | *Chenonetta jubata* |
| White-faced heron | *Ardea novaehollandiae* |
| Australian white ibis | *Threskiornis aethiopica* |
| Black-winged stilt | *Himantopus himantopus* |
| Pacific heron | *Ardea pacifica* |
| Wandering whistling-duck | *Dendrocygna arcuata* |
| Yellow-billed spoonbill | *Platalea flavipes* |

^a^ Species that could not be separated during aerial surveys.
